# Role of Acetylsalicylic Acid in Cytokine Stimulation of Macrophages in Antibody-Dependent Cellular Cytotoxicity (ADCC)

**DOI:** 10.1155/S0962935194000591

**Published:** 1994

**Authors:** M. Jäpel, H. Lötzerich, K. Rogalla

**Affiliations:** 1 Institute for Experimental Morphology German Sports University P.O. Box 45 03 27 Köln 50927 Germany; 2 Drug Research Center Bayer AG Aprather Weg Wuppertal 1 5600 Germany

## Abstract

In addition to the spectrum of biological action already known to be exhibited by acetylsalicylic acid (ASA) as an analgesic, anti-inflammatory and platelet aggregation inhibitor, there is growing evidence of a stimulatory effect on the immune system. ASA has been found to increase the production ofcytokines and to increase the activity of various leukocytes. The action of ASA on the activity of mouse peritoneal macrophages was therefore investigated in the present study. Therapeutically effective concentrations of ASA, which are known to decrease levels of prostaglandins, had neither a stimulating nor an inhibiting influence on antibody-dependent cellular cytotoxicity (ADCC) or on the binding capacity of macrophages with regard to SW 948 tumour cells. Likewise ASA had little or no adverse effect on the capacity of the macrophages for stimulation by interferon-gamma (IFN-gamma) and interleukin-4 (IL-4). Taken together, the immunostimulant effect of ASA shown in the literature as an increased production of interleukin-2 (IL-2) and IFN, could not be confirmed on the basis of the macrophage cytotoxiclty.


Mediators of Inflammation 3, 419–424 (1994)

### Role of acetylsalicylic acid in cytokine stimulation of macrophages in antibody-dependent cellular cytotoxicity (ADCC)

M. Jäpel,[1] H. Lötzerich[1,CA] and K. Rogalla[2]

[1]Institute for Experimental Morphology, German Sports University, P.O. Box 45 03 27, 50927 Köln, Germany; [2]Drug Research Center, Bayer AG, Aprather Weg, 5600 Wuppertal 1, Germany

[CA] Corresponding Author

**IN addition to the spectrum of biological action already known to be exhibited by acetylsalicylic acid (ASA) as an analgesic, anti-inflammatory and platelet aggregation inhibitor, there is growing evidence of a stimulatory effect on the immune system. ASA has been found to increase the production of cytokines and to increase the activity of various leukocytes. The action of ASA on the activity of mouse peritoneal macrophages was therefore investigated in the present study. Therapeutically effective concentrations of ASA, which are known to decrease levels of prostaglandins, had neither a stimulating nor an inhibiting influence on antibody-dependent cellular cytotoxicity (ADCC) or on the binding capacity of macrophages with regard to SW 948 tumour cells. Likewise ASA had little or no adverse effect on the capacity of the macrophages for stimulation by interferon-gamma (IFN-gamma) and interleukin-4 (IL-4). Taken together, the immunostimulant effect of ASA shown in the literature as an increased production of interleukin-2 (IL-2) and IFN, could not be confirmed on the basis of the macrophage cytotoxicity.**

**Key words:** Acetylsalicylic acid, ADCC, Biological response modifiers, Macrophages

### Introduction

Many lines of experimental evidence suggest that acetylsalicylic acid (ASA) is able to act as an immunomodulating agent. ASA increases the production of interleukin-2 (IL-2) and interferon (IFN).[1–7] On oral administration of ASA, synthesis of IL-2 by peripheral mononuclear blood cells reaches its peak after 10 h and the synthesis of IFN-gamma is greatest after 24 h. On stimulation with ASA *in vitro* the peaks of IL-2 and IFN-gamma synthesis occur somewhat later, i.e. after 24 h and 72 h respectively. Moreover, cytokine synthesis is dependent upon the presence of monocytes, as no effect was observable in isolated lymphocytes cultures.[4,7] The time of occurrence of the IL-2 and IFN-gamma maxima thus corresponds to the model of cytokine regulation[2] and underlines the importance of the monocytes and macrophages in this activation of the immune system. This provides further evidence of the scientific basis of the immunological effect of ASA, which can be explained in terms of inhibition of prostaglandin synthesis by monocytes and macrophages.[1,2,4,8,9] In this reaction ASA inhibits cyclooxygenase activity irreversibly by covalent binding of its acetyl group to the enzyme.[10] Among the arachidonic acid metabolites the prostaglandins of group E (PGE) exert a suppressive effect on the immune system.[11] The proliferation of T-lymphocytes, lymphokine production, and the cytotoxicity of Nk cells, lymphocytes and macrophages are inhibited by PGE,[12–18] while tumour growth[15,19,20] and metastatic growth[21–23] are promoted. The depression of the immune system can therefore be explained on the basis of elevated PGE production or by increased sensitivity to PGE. Conversely, PGE-synthesis inhibitors act as immunostimulants. PGE-synthesis blockers reduce or slow down tumour growth.[19,24–26] ASA also enhances the cytotoxicity of Nk cells in tumour-bearing animals.[27] There is also an epidemiological study according to which ASA exerts a protective effect against cancer of the colon; regular intake of ASA was found to reduce the risk of colon cancer significantly both in men and in women.[28] Since the activation of macrophages plays an important part in tumour defence,[29–31] and the macrophages are directly affected by the inhibition of PGE synthesis, the aim of the present study was to determine the extent to which ASA influences the activation of macrophages by IL-4 and IFN-gamma.

### Materials and Methods

*Mice*: Female, syngenic C57Bl/6 mice, 8–12 weeks of age, were purchased from IFFA Credo (Saint-Germain-Sur-L'Arbesle, France) and were matched for age in each experiment. The animals were housed conventionally in plastic cages and were given water and food *ad libitum.*

© 1994 Rapid Communications of Oxford Ltd

*Cell line*: The SW 948 colonic adenocarcinoma cell line was established by Leibovitz *et al.*[32] and was kindly provided by H. Löhrke (German Cancer Center (DKFZ), Heidelberg, Germany). The cell line was maintained in Leibovitz's L-15 medium containing 10% foetal bovine serum, 2 mM glutamine, 100 U/ml penicillin/streptomycin and 2.5 μg/ml fungizone (all ICN-Flow, Germany). The tumour cells were cultured in 75 $cm^2$ plastic tissue flasks and passed weekly.

*Cytokines*: Interleukin-4 ($3 \times 10^4$ U/ml) was supplied by Genzyme (Germany). Recombinant IFN-gamma ($1 \times 10^5$ U/ml) was purchased from Boehringer (Germany). Both cytokines were diluted in PBS supplement with 0.1% bovine serum albumin, aliquotted and stored at –80°C until used.

*Polyclonal antibodies*: Anti-SW 948 serum was prepared in C57BL/6 mice as follows, according to the method of Johnson *et al.*:[33] in a first step mice received an i.p. injection of $10^6$ tumour cells in 0.1 ml Hank's buffered salt solution (HBSS). Two weeks later, in a second step, the mice received an i.p. injection of $10^6$ tumour cells in HBSS. Ten days after the final injection 2–3 ml blood was collected by cardiac puncture. Serum was separated after centrifugation and 50 μl aliquots were stored at –80°C until use. The antisera alone were not capable of causing tumour cell lysis.

*Acetylsalicylic acid*: The acetylsalicylic acid was purchased from Bayer (Germany) in the form of the lysine salt (Aspisol$^R$). It was dissolved under sterile conditions in distilled water for injections. It is very important to prepare the solution of Aspisol immediately before use, because hydrolysis of acetylsalicylic acid sets in very quickly in aqueous solutions.

*Harvest of peritoneal macrophages*: Mice were killed by cervical dislocation and proteose peptone- and thioglycollate-elicited macrophages were harvested 72 h after injection of 0.6 ml of each agent by peritoneal lavage. Eight ml of cold HBSS containing 10 U/ml heparin was injected into the peritoneal cavity of the mice and peritoneal exudate cells were harvested. The cell suspensions were centrifuged at 500 × **g** for 5 min. The cells were resuspended in minimal essential medium (MEM; Gibco, Germany) supplemented as above and a small sample was taken for total and differential cell counts. The thioglycollate treatment leads to an over 95% macrophage content of the peritoneal exudate cells, in contrast to peptone-elicited macrophages (65%). The peritoneal exudate cells were added to 96-well flat-bottom plates (Bibby, UK) at the desired macrophage concentrations and were incubated at 37°C in a humidified atmosphere of 5% $CO_2$. After 1 h of incubation the nonadherent cells were washed off, obtaining a monolayer with more than 98% macrophages.[34] The macrophages were now ready for use in the antibody-dependent cellular cytotoxicity (ADCC) or binding assays. In experiments with IFN all macrophages were cultured for the duration of 48 h. Two hours of stimulation with IFN or ASA indicates the period before starting the ADCC or binding assays. In costimulation with IL-4 all macrophages were cultured and stimulated over a 24 h period before starting the assays.

*ADCC*: The slow form of ADCC was estimated as previously described.[35] In brief, $4 \times 10^4$ SW 948 tumour cells labelled with [$^3$H]thymidine (TRK. 120, sp. act. 25 μCi/mmol, Amersham Buchler, Braunschweig, Germany) were added to the monolayers of macrophages ($1 \times 10^5$ per well) in the 96-well flat-bottom plates either with or without the polyclonal anti-SW cell antiserum (Ab). The plates were harvested after an incubation period of 48 h at 37°C in a humidified atmosphere of 5% $CO_2$. A cell-free supernatant (100 μl) was removed and added to scintillation cocktail (Canberra Packard, Frankfurt, Germany). The ADCC was quantified using the relationship:

$$\%\ \text{Lysis} = \frac{\begin{array}{c}(\text{cpm released in tests with Ab—spont. rel.})\\ -(\text{cpm released in tests without Ab—spont. rel.})\end{array}}{\text{cpm total releaseable (=maximum release)}} \times 100$$

All tests were carried out in triplicate and repeated three times.

*Binding assay*: The estimation of the binding capacity of macrophages to tumour cells was performed as previously described in detail.[36] In brief, $4 \times 10^4$ SW 948 tumour cells labelled with [$^3$H]thymidine (TRK.120, sp. act. 25 μCi/mmol, Amersham Buchler, Braunschweig, Germany) were added to the monolayers of macrophages ($1 \times 10^5$ per well) in the 96-well flat-bottom plates either in the presence or in the absence of the polyclonal anti-SW cell antiserum (Ab). After centrifugation at 50 × **g** for 1 min and an incubation time of 15 min unbound tumour cells were completely removed by aspiration and four vigorous washings with HBSS. The remaining bound target cells were lysed by adding 200 μl of 0.25% sodium dodecyl sulphate (Sigma, Germany) to each well. Binding was quantified using the relationship:

$$\text{No. of bound target} = \frac{\text{cpm bound to macrophages}}{\text{total cpm added}} \times 4 \times 10^4$$

All tests were carried out fourfold and repeated three times.

*Statistics*: Experimental results were analysed for significant differences between points at confidence level $p < 0.05$ by analysis of variance.

### Results

The ASA concentrations studied (75 μg/ml and 100 μg/ml) did not have any inhibiting or stimulating effect on the cytotoxic activity of the peptone-elicited macrophages (Figs 1 and 2). This is clear both from the antibody-independent and from the antibody-dependent cytotoxic capacity of the macrophages with respect to the tumour cells. Accordingly, the ADCC value was also unchanged. By contrast, IL-4 concentrations of 10 and 20 U/ml were found to activate the peptone-elicited macrophages to the level of the thioglycollate macrophages. Similarly, costimulation of the macrophages with IL-4 and ASA induced a significant increase in macrophage activity. A weak inhibition of macrophage activity was observable after costimulation in comparison with stimulation exclusively with IL-4, though this was not significant (Figs 1 and 2).

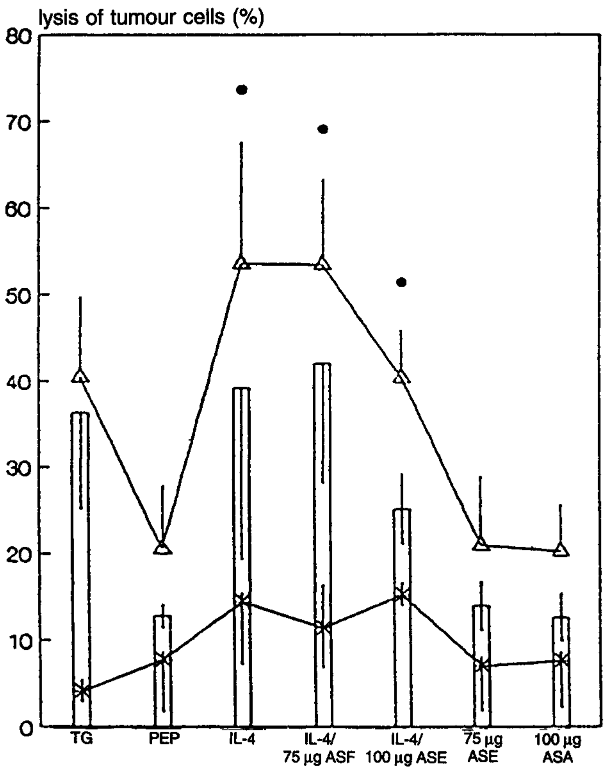

FIG. 1. Results of ASA costimulation with IL-4 in tumour cell lysis. Peritoneal macrophages (MP) from mice injected with proteose peptone (PEP) were prepared at 1 × $10^5$ MP/well. After nonadherent cells were removed, 200 μl MEM+ with either 75 or 100 μg ASA/ml with or without 10 U IL-4/ml were added. After 24 h the total volume was replaced by 200 μl fresh medium with 4 × $10^4$ [$^3$H]thymidine-labelled SW 948 cells, and with or without antibodies (Ab). After 48 h, 100 μl supernatant was aspired from each sample and cpm were determined. The cell lysis with Ab (△), the cell lysis without Ab (*), and the ADCC (bars) were calculated as described in the text. Thioglycollate (TG) elicited MPs served as control, only. (●) Significantly different to PEP-control.

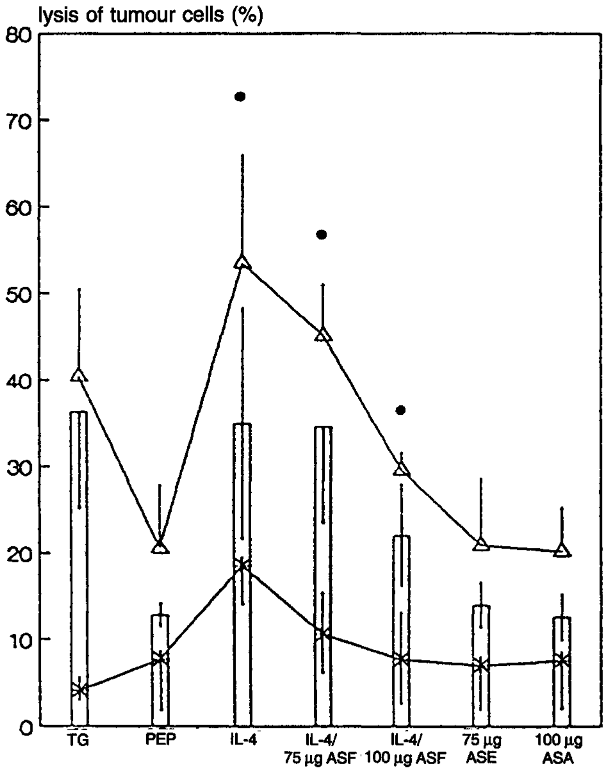

FIG. 2. Results of ASA costimulation with IL-4 in tumour cell lysis. Peritoneal macrophages (MP) from mice injected with protease peptone (PEP) were prepared at 1 × $10^5$ MP/well. After nonadherent cells were removed, 200 μl MEM+ with either 75 or 100 μg ASA/ml with or without 20 U IL-4/ml were added. After 24 h the total volume was replaced by 200 μl fresh medium with 4 × $10^4$ [$^3$H]thymidine-labelled SW 948 cells, and with or without antibodies (Ab). After 48 h, 100 μl supernatant was aspired from each sample and cpm were determined. The cell lysis with Ab (△), the cell lysis without Ab (*), and the ADCC (bars) were calculated as described in the text. Thioglycollate (TG) elicited MPs served as control, only. (●) Significantly different to PEP-control.

Whereas the binding capacity of the macrophages with respect to the tumour cells without antibodies was found to be uninfluenced in all reactions, the antibody-assisted binding capacity of the thioglycollate-elicited macrophages showed a significant increase. Macrophage stimulation with IL-4 and ASA or a combination of the two led to a slight, but not significant, increase in bonds (Figs 3 and 4).

Stimulation of the peptone-elicited macrophages with 100 U IFN-gamma/ml significantly increased the ADCC value and tumour toxicity in the presence of antibodies in comparison with controls (Fig. 5). In contrast, no significant increase in macrophage activity was observed on costimulation with 75 μg ASA/ml. As a result of the reduction in antibody-dependent tumour toxicity, this value was found to be between that of the peptone controls and that after activation with IFN-gamma, without differing significantly from either of these (Fig. 5).

The binding capacity of the macrophages was affected neither positively nor negatively by IFN-gamma and/or ASA (Fig. 6).

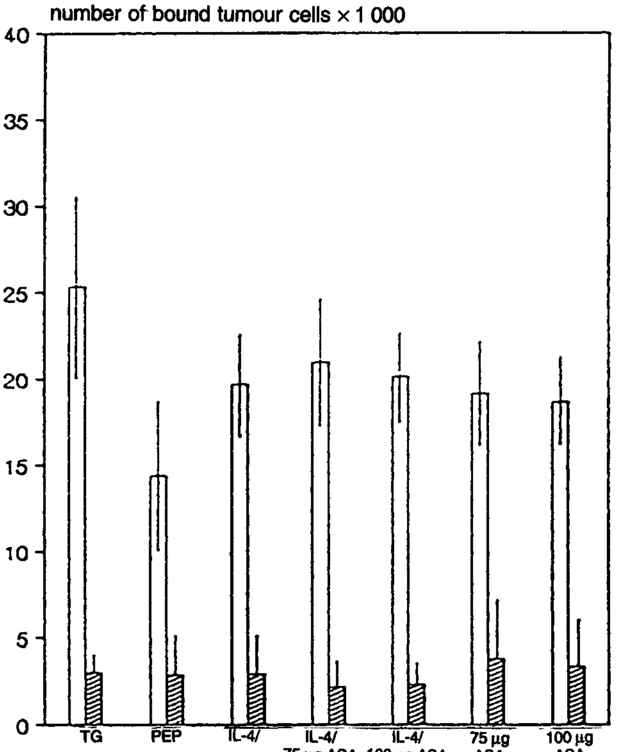

FIG. 3. Results of ASA costimulation with IL-4 on bound tumour cells. Peritoneal macrophages (MP) from mice injected with protease peptone (PEP) were prepared at 1 × $10^5$ MP/well. After nonadherent cells were removed, 200 µl MEM+ with 75 or 100 µg ASA with or without 10 U IL-4/ml were added. After 24 h the total volume was replaced with 200 µl fresh medium with 4 × $10^4$ [$^3$H]thymidine-labelled SW 948 cells, and with or without antibodies (Ab). After 15 min, the total volume was aspired from each sample and cpm were determined. The number of bound tumour cells with Ab (clean bars) or without Ab (striped bars) were calculated as described in the text. Thioglycollate (TG) elicited macrophages served as control only.

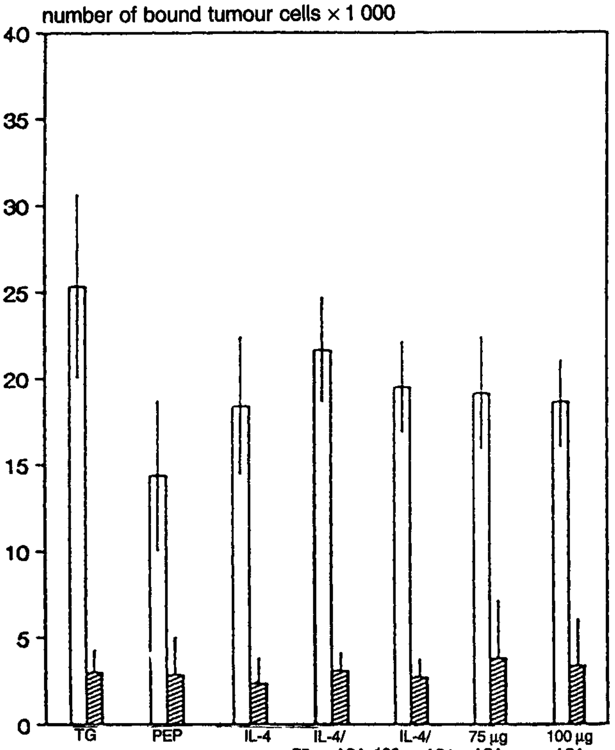

FIG. 4. Results of ASA costimulation with IL-4 on bound tumour cells. Peritoneal macrophages (MP) from mice injected with protease peptone (PEP) were prepared at 1 × $10^5$ MP/well. After nonadherent cells were removed, 200 µl MEM+ with 75 or 100 µg ASA with or without 20 U IL-4/ml were added. After 24 h the total volume was replaced with 200 µl fresh medium with 4 × $10^4$ [$^3$H]thymidine-labelled SW 948 cells, and with or without antibodies (Ab). After 15 min, the total volume was aspired from each sample and cpm were determined. The number of bound tumour cells with Ab (striped bars) or without Ab (striped bars) were calculated as described in the text. Thioglycollate (TG) elicited macrophages served as control only.

### Discussion

A comparison of our results reveals a consistent tendency of ASA to have no essential effect on the activity of murine peritoneal macrophages *in vitro*. Macrophage activation, such as that induced by IL-4 and IFN-gamma, was not observed, and there was also no synergism between ASA and the cytokines. No increase or inhibition of macrophage activity was observed, although slight inhibition occurred in the experiments with costimulation with IFN-gamma. The immunostimulant effect of ASA reported in the literature could not be confirmed on the basis of the ADCC model. The inhibition of prostaglandin synthesis by the therapeutically effective concentrations of ASA used might play a role as a possible explanation for this *in vivo*, since prostaglandins, and especially PGE, are among the inhibitory immunomediators within the immune regulation system and are responsible for a number of immunosuppressive mechanisms at the level of cellular immunity.[15,37–39] It is therefore likely that prostaglandin synthesis inhibitors can act as immunostimulants. Thus, cyclooxygenase inhibition leads to increased macrophage activity, manifested by an increased production of IL-1.[40] In turn, IL-1 leads to a stimulation of T- and B-lymphocytes.[41] The elevated synthesis rates of IL-2 and IFN-gamma after administration of ASA can therefore be explained by an interaction of macrophages and lymphocytes with their mutual activation.[3–5]

The need for the presence of macrophages is indicative of primary stimulation of the macrophages by ASA. No such immunostimulant action of ASA on isolated peritoneal macrophages was detected in this study. The therapeutic concentrations of ASA achievable in human blood (75–100 µg/ml) have no direct influence on macrophages under *in vitro* conditions. This applies both to the binding capacity and to the antibody-dependent and -independent tumour toxicity of mouse peritoneal macrophages. The ADCC of macrophages is likewise not changed in either direction by ASA, even though prostaglandins suppress ADCC and the tumour-toxic activity of macrophages.[12–15] In the selected experimental design this effect evidently does not come into operation. Our findings are therefore in agreement with the results obtained by Hockertz *et al.*,[42] who were

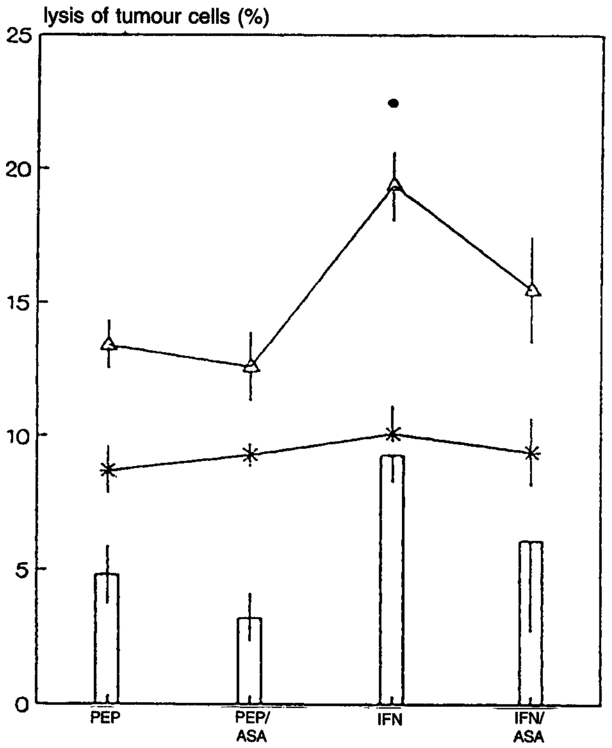

FIG. 5. Effect of ASA costimulation with IFN-gamma on tumour cell lysis. Peritoneal macrophages (MP) from mice injected with protease peptone (PEP) were prepared at 1 × 10$^5$ MP/well. Two h before starting the ADCC medium was aspired and replaced with fresh MEM$^+$ containing either 75 μg ASA/ml or 100 U IFN-gamma/ml or both, respectively. After the appropriate period of time, medium was aspired and 200 μl medium with 4 × 10$^4$ [$^3$H]thymidine-labelled SW 948 cells with or without antibodies (Ab) were added to each well. After 48 h, 100 μl were aspired from each sample and cpm were determined. The cell lysis with Ab (△), the cell lysis without Ab (*), and the ADCC (bars) were calculated as described in the text. IFN-gamma significantly increases the ADCC in correspondence to cell lysis with Ab. ASA reduces the effect of IFN-gamma. (●) Significantly different to PEP-control.

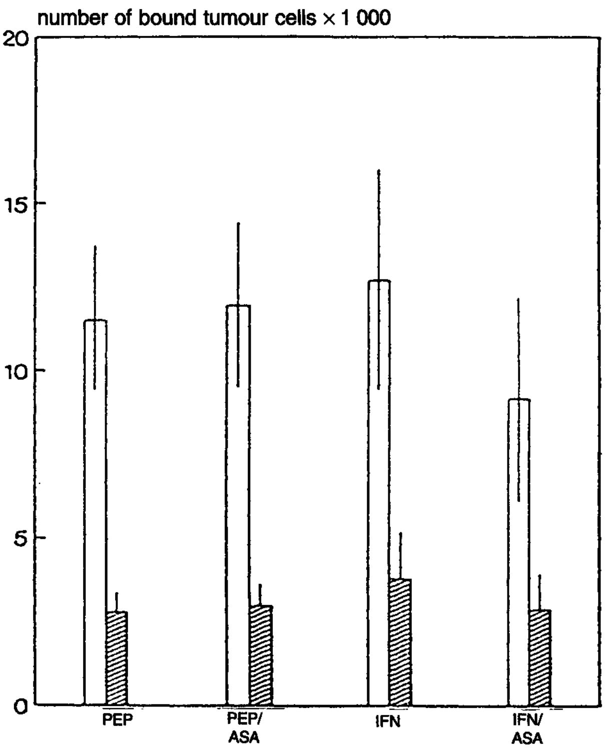

FIG. 6. Effect of ASA costimulation with IFN-gamma on tumour cell binding. Peritoneal macrophages (MP) from mice injected with protease peptone (PEP) were prepared at 1 × 10$^5$ MP/well. Two h before starting the binding assay medium was aspired and replaced with fresh MEM$^+$ containing either 75 μg ASA/ml or 100 U IFN-gamma/ml or both, respectively. After the appropriate period of time, medium was aspired and 200 μl medium with 4 × 10$^4$ [$^3$H]thymidine-labelled SW 948 cells with or without Ab were added to each well. After 15 min, the total volume was aspired from each sample and cpm were determined. The number of bound tumour cells with Ab (clean bars) or without Ab (striped bars) were calculated as described in the text.

likewise unable to observe any effect of ASA on isolated murine peritoneal macrophages. In addition to the unchanged production of IL-6, the production of oxygen radicals also remained unchanged, although these play an important part as tumour-toxic effector substances of the macrophages precisely in the ADCC reaction.[43–45]

The stimulation of the macrophages by the cytokines IFN-gamma and IL-4 corresponds to the findings of other studies.[31,46–48] A slight change in macrophage activity was observed only on costimulation with IFN-gamma and ASA. In this case the antibody-dependent cytotoxicity and the ADCC values were inhibited by costimulation with ASA. It may be that costimulation causes an increase in the cAMP level, which exerts an inhibitory effect on the macrophages and their ADCC activity.[49,50] By contrast ASA exhibits neutral behaviour in tumour patients receiving IFN.[51] There is no synergistic effect between stimulant cytokines and ASA.

Looking at the results overall, the antibody-dependent and -independent tumour toxicity and the binding capacity of peritoneal macrophages are not directly influenced *in vitro* by the presence of ASA. The positive immunological influence of ASA is therefore very probably closely connected with an interaction between defence cells and their cytokines. It is still unclear to what extent ASA can be used as a direct immunostimulant or immunomodulator, but its clinical use, e.g. as an adjuvant in inoculations, could be of major importance.

### References

1. Zatz MM, Skotnicki A, Bailey JM, Oliver JH, Goldstein AL. Mechanism of action of thymosin. II. Effects of aspirin and thymosin on enhancement of IL-2 production. *Immunopharmacology* 1985; **9:** 189–198.
2. Yousefi S, Chiu J, Carandang G, Archibeque EG, Vaziri N, Cesario TC. Effect of acetylsalicylic acid on reduction and action of leukocyte-derived interferons. *Antimicrob Agents Chemother* 1987; **31:** 114–116.
3. Cesario TC, Yousefi S, Carandang G. The regulation of interferon production by aspirin, other inhibitors of the cyclooxygenase pathway and agents influencing calcium channel flux. *Bull NY Acad Med* 1989; **65:** 26–35.
4. Hsia J, Sarin N, Oliver JH, Goldstein AL. Aspirin and thymosin increase interleukin-2 and interferon-gamma production by human peripheral blood lymphocytes. *Immunopharmacology* 1989; **17:** 167–173.
5. Hsia J, Simon GL, Higgins N, Goldstein AL, Hayden FG. Immune modulation by aspirin during experimental rhinovirus colds. *Bull NY Acad Med* 1989; **65:** 45–56.
6. Pugliese A, Maggiolo F. Torre D, Forno B, Pollono AM, Martelli ML, Biglino A. Influence of acetylsalicylic acid and of salicylic acid on mediators of immune response. *Int J Immunotherapy* 1991; **7:** 31–35.

7. Hsia J, Tang T. Aspirin a biological response modifier. In: Goldstein AL, Garaci E, eds. *Combination Therapies*. New York: Plenum Press, 1992; 131–137.
8. Juan H. Kürzlich entdeckte Wirkungsmechanismen der nichtsteroidalen Antirheumatika: Mögliche, nicht nur symptomatische therapeutische Effekte. *Wiener Klin Wochenschr* 1983; **95:** 865–869.
9. Rumore MM, Aron SM, Hiross EJ. A review of mechanism of action of aspirin and its potential as an immunomodulating agent. *Med Hypotheses* 1987; **22:** 387–400.
10. Roth GJ, Siok CJ. Acetylation of the $NH_2$-terminal serine of prostaglandin synthetase by aspirin. *J Biol Chem* 1978; **253:** 3782–3784.
11. Waymack JP. Effect of prostaglandin E on immune function in multiple animal models. *Arch Surg* 1988; **123:** 1429–1432.
12. Droller MJ, Schneider MU, Perlmann P. A possible role of prostaglandins in the inhibition of natural and antibody-dependent cell-mediated cytotoxicity against tumor cells. *Cell Immunol* 1978; **39:** 165–177.
13. Trofatter KF, Daniels A. Interaction of human cells with prostaglandins and cyclic AMP modulators. *J Immunol* 1979; **122:** 1363–1370.
14. Taffet SM, Russell SW. Macrophage-mediated tumor cell killing: regulation of expression of cytolytic activity by prostaglandin E. *J Immunol* 1980; **126:** 424–427.
15. Goodwin JS, Ceuppens J. Regulation of the immune response by prostaglandins. *J Clin Immunol* 1983; **3:** 295–315.
16. Schenkelaars EJ, Bonta IL. Cyclooxygenase inhibitors promote the leukotriene $C_4$ induced release of beta-glucuronidase from rat peritoneal macrophages: prostaglandin $E_2$ suppresses. *Int J Immunopharmac* 1986: **8:** 305–311.
17. Voth R, Chmielarczyk W, Storch E, Kirchner H. Induction of natural killer cell activity in mice by injection of indomethacin. *Nat Immun Cell Growth Regul* 1986; **5:** 317–324.
18. Field WE, Ferguson FG, Reddanna P, Reddy CC. The effect of selected arachidonic acid metabolites on natural killer cell activity. *Prostaglandins* 1988; **36:** 411–419.
19. Plescia OJ, Smith AH, Ginwich K. Subversion of immune system by tumor cells role of prostaglandins. *Proc Natl Acad Sci USA* 1975; **72:** 1848–1851.
20. Lupulescu A. Enhancement of carcinogenesis by prostaglandins. *Nature* 1978; **272:** 634–636.
21. Young MR, Newby M. Differential induction of suppressor macrophages by cloned Lewis lung carcinoma variants in mice. *JNCI* 1986; **77:** 1255–1260.
22. Young MR, Wheeler E, Newby M. Macrophage-mediated suppression of natural killer cell activity in mice bearing Lewis lung carcinoma. *JNCI* 1986; **76:** 745–750.
23. Young MR, Newby M. Enhancement of Lewis lung carcinoma cell migration by prostaglandin $E_2$ produced by macrophages. *Cancer Res* 1986; **46:** 160–164.
24. Hial V, Horakova Z, Shaff R, Beaven MA. Alteration of tumor growth by aspirin and indomethacin: studies with two transplantable tumors in mouse. *Eur J Pharmacol* 1976; **37:** 367–376.
25. Lynch NR, Castes M, Astoin M, Salomon JC. Mechanism of inhibition of tumor growth by aspirin and indomethacin. *Br J Cancer* 1978; **38:** 503–512.
26. Valdez JC, Perdigon G. Piroxicam, indomethacin and aspirin action on a murine fibrosarcoma. Effects on tumor-associated and peritoneal macrophages. *Clin Exp Immunol* 1991; **86:** 315–321.
27. Brunda MJ, Herberman RB, Holden HT. Inhibition of murine natural killer cell activity by prostaglandins. *J Immunol* 1980; **124:** 2682–2687.
28. Thun MJ, Namboodiri MM, Heath CW. Aspirin use and reduced risk of colon cancer. *New Engl J Med* 1991; **325:** 1593–1596.
29. Adams DO, Hall T, Steplewski Z, Koprowski H. Tumors undergoing rejection by monoclonal antibodies of IgG 2a isotype contain increased numbers of macrophages activated for a distinctive form of antibody-dependent cytolysis. *Proc Natl Acad Sci USA* 1984; **81:** 3506–3510.
30. Weiner LM, Steplewski Z, Koprowski H, Sears HF, Litwin S, Comis RL. Biological effects of gamma interferon pre-treatment followed by monoclonal antibody 17-1A administration in patients with gasatrointestinal carcinoma. *Hybridoma* 1986; **5:** S65–S77.
31. Fan S, Fehr HG, Adams DO. Activation of macrophages for ADCC *in vitro*: effects of IL-4, TNF, interferons alpha/beta, interferon-gamma, and GM-CSF. *Cell Immunol* 1991; **135:** 78–87.
32. Leibovitz A, Stinson WB, McCombs WB, McCoy CE, Mazur KC, Maybry ND. Classification of human colorectal adenocarcinoma cell lines. *Cancer Res* 1976; **36:** 4562–4569.
33. Johnson WJ, Bolognesi DP, Adams DO. Antibody-dependent cytolysis (ADCC) of tumor cells by activated murine macrophages is a two-step process: quantification of target binding and subsequent target lysis. *Cell Immunol* 1984; **83:** 170–180.
34. Adams DO. Quantitation of adherent mononuclear phagocytes by inverted phase microscopy. In: Adams DO, Edelson PE, Koren H, eds. *Methods for Studying Mononuclear Phagocytes*. New York: Academic Press, 1981; 325–330.
35. Adams DO, Hamilton TA. Phagocytic cells. Cytotoxic activities of macrophages. In: Gallin JJ, Goldstein JM, Snyderman R, eds. *Inflammation: Basic Principles and Clinical Correlates*. New York: Raven Press, 1988; 471–491.
36. Johnson WJ, Adams DO. Assays detecting the antibody-dependent and independent binding and cytolysis of tumor cells by murine macrophages. In: Colowick S, Plam NO, eds. *Methods in Enzymology, Vol 132*. New York: Academic Press, 1986; 555–568.
37. Goodwin JS, Webb DR. Regulation of immunoresponse by prostaglandins. *Clin Immunol Immunopath* 1980; **15:** 106–122.
38. Rappaport RS, Dodge GR. Prostaglandin E inhibits the production of human interleukin 2. *J Exp Med* 1982; **155:** 943–948.
39. Chouaib S, Chatenould L, Klatzmann D, Fradelizi D. The mechanisms of inhibition of human IL 2 production. II. $PGE_2$ induction of suppressor T lymphocytes. *J Immunol* 1984; **133:** 1851–1857.
40. Kunkel SL, Chensue SW, Phan SH. Prostaglandins as endogenous mediators of interleukin 1 production. *J Immunol* 1986; **136:** 186–192.
41. Dinarello CA. Interleukin-1 and the effects of cyclooxygenase inhibitors on its biological activities. *Bull NY Acad Med* 1989; **65:** 80–92.
42. Hockertz S, Schettler T, Rogalla K. Effect of acetylsalicylic acid, ascorbate and ibuprofen on the macrophage system. *Arzneim Forsch Drug Res* 1992; **42:** 1062–1068.
43. Nathan CF. Reactive oxygen intermediates in lysis of antibody-coated tumor cells. In: Koren HS, ed. *Macrophage-mediated Antibody-dependent Cellular Cytotoxicity*. New York: Dekker, 1983; 199–216.
44. Adams DO, Hamilton TA. Destruction of tumor cells by mononuclear phagocytes: models of analysing effector mechanisms and regulation of macrophage activation. In: North RJ, Steinman RM, eds. *Basic Mechanisms of Host Resistance to Infectious Agents, Tumors and Allografts*. New York: Rockefeller University Press, 1986: 185–204.
45. Adams DO. Molecular interactions in macrophage activation. *Immunol Today* 1989; **10:** 33–35.
46. Somers SD, Erickson KL. Regulation of murine macrophage function by IL-4. I. Activation of macrophages by a T–T cell hybridoma is due to IL-4. *Cell Immunol* 1989; **22:** 178–187.
47. Nacy A, Meltzer MS. T-cell-mediated activation of macrophages. *Current Opinion Immunol* 1991; **3:** 330.
48. Wersäll P, Masucci G, Mellstedt H. Interleukin-4 augments the cytotoxic capacity of lymphocytes and monocytes in antibody-dependent cellular cytotoxicity. *Cancer Immunol Immunother* 1991; **33:** 45–49.
49. Garovoy MR, Strom TB, Kaliner M, Carpenter CB. Antibody-dependent lymphocyte mediated cytotoxicity mechanism and modulation by cyclic nucleotides. *Cell Immunol* 1975; **20:** 197–204.
50. Okonogi K, Gettys TW, Uhing RJ, Tarry WC, Adams DO, Prpic V. Inhibition of prostaglandin E2-stimulated cAMP accumulation by lipopolysaccharide in murine peritoneal macrophages. *J Biol Chem* 1991; **266:** 10305–10312.
51. Creagan ET, Twito DI, Johansson SL, Schaid DJ, Johnson PS, Flaum MA, Buroker TR, Geeraerts LH, Veeder MH, Gesme DH, Homburger HA. A randomized prospective assessment of recombinant leukocyte A human interferon with or without aspirin in advanced renal adenocarcinoma. *J Clin Oncol* 1991; **9:** 2104–2109.

ACKNOWLEDGEMENTS. We thank Mrs M. Euler and Mrs M. Maskus for their excellent technical assistance, and Mrs S. Becker for her patient secretarial services.

**Received 1 March 1994; accepted in revised form 15 July 1994**
